# Whole Genome Sequencing and Comparative Genomics Analysis of Goat-Derived *Klebsiella oxytoca*

**DOI:** 10.3390/genes16010013

**Published:** 2024-12-26

**Authors:** Yu Zhang, Zhenxing Zhang, Ziying Wang, Yimei Chen, Lianjie Liao, Li Du, Hongyan Gao, Qiaoling Chen, Churiga Man, Si Chen, Fengyang Wang

**Affiliations:** 1 Hainan Key Lab of Tropical Animal Reproduction, Breeding and Epidemic Disease Research, Animal Genetic Engineering Key Lab of Haikou, Hainan University, Haikou 570228, China; 2 School of Tropical Agriculture and Forestry, Hainan University, Haikou 570228, China

**Keywords:** *Klebsiella oxytoca*, genome sequencing, virulence-related genes, comparative genomics analysis

## Abstract

**Background:** This research aims to enhance the genomic database of *Klebsiella oxytoca* by identifying virulence genes through the whole genome sequencing and comparative analysis of a goat-derived *K. oxytoca* (KOHN1) strain, while clarifying the relationship between its genetic evolution and virulence, ultimately providing a theoretical foundation for clinical prevention and diagnosis. **Methods:** Third-generation Oxford Nanopore Technologies (ONT) sequencing and second-generation Illumina sequencing were used to sequence the strain and analyze the database annotations. Screening for 10 virulence genes was conducted using PCR. Comparative genomic analyses of the strain KOHN1 with four human-derived *K. oxytoca* model strains were performed using collinearity analysis, taxonomy classification through ANI analysis, and gene function family analysis. **Results:** The genome size of the KOHN1 strain was 5,817,806 bp, and the GC content was 55.14%. It contained 5227 predicted coding genes, including 25 rRNA genes, 85 tRNA genes, and 53 sRNA genes. A total of 14 type VI secretion system effector proteins and 146 virulence factor-related genes were annotated. Additionally, eight virulence genes—*fimA*, *fimH*, *entB*, *mrkD*, *clpV*, *rmpA*, *vgrG,* and *hcp*—were detected through PCR identification. The strain has 448 drug resistance genes, mainly against β-lactams and fosfomycins. Comparative genomic analysis indicated that its closest relation is the human isolate ASM338647. **Conclusions:** In this study, the whole genome sequence of a goat-derived *K. oxytoca* (KOHN1) strain was obtained, revealing its evolutionary relationship with domestic and foreign isolates and providing a reference for future studies on the mechanisms of antimicrobial resistance and the pathogenicity of *K. oxytoca*.

## 1. Introduction

*K. oxytoca* belongs to the genus *Klebsiella* of Enterobacteriaceae. It is a Gram-negative opportunistic pathogen that can cause zoonotic infectious diseases and is the second most important clinical species of *Klebsiella* after *K. pneumoniae* [1]. *K. oxytoca*, a zoonotic pathogen, is capable of causing multiple types of infections, such as antibiotic-associated hemorrhagic colitis (AAHC), urinary tract infections, and bacteremia [2]. It mainly infects the intestines by producing enterotoxins during the infant period of animals, resulting in complications such as bacteremia [3]. These complications may ultimately lead to significant morbidity and mortality in both humans and animals [4,5]. At present, the potential transmission of this pathogen and its associated drug resistance genes from animals to humans is a pressing public health concern [6].

In recent years, the misuse of antibiotics has led to an increase in antibiotic resistance (AMR), particularly to β-lactams [7], fosfomycin, and fluoroquinolones [8]. This has resulted in treatment failures in many cases and the emergence of multi-drug-resistant strains, which have serious economic implications for goat farms. As a multi-drug-resistant bacterium, studies indicate that *K. oxytoca* may exhibit greater resistance than the more commonly encountered *K. pneumoniae*. Furthermore, infections caused by *K. oxytoca* isolates demonstrate higher invasiveness compared to those caused by *K. pneumoniae* isolates [9]. Therefore, although the prevalence of *K. oxytoca* is low, it causes more serious invasive infections and is more harmful to humans and animals. This finding may be related to the increased frequency of specific virulence factors.

At present, the main virulence genes related to *K. oxytoca* include *wabG*, *fimA*, *fimH*, *mrkD*, *ureA*, and others [10]. Lipopolysaccharide-related genes, such as *wabG*, are crucial virulence factors in *K. pneumoniae* that contribute to the development of invasive infections [11]. Adhesin and fimbrial genes of type 1 (*fim*) and type 3 (*mrk*) are also important factors in the pathogenicity of *Klebsiella*. Currently, research into the infection mechanisms of *K. oxytoca* remains in the exploratory phase. It is believed that the early immune response in the respiratory tract to *K. oxytoca* exhibits specific characteristics and corresponding immune reactions, which may be related to a microbial imbalance [12]. *K. oxytoca* produces adhesins that enhance interactions between the bacteria and host cells, as well as with other microbial species [13]. In addition, the study found that *K. oxytoca* was positively correlated with the *in vitro* growth of *Clostridium* difficile and negatively correlated with *Staphylococcus aureus*. The interaction of multiple pathogens during infection may be the cause of the complex pathogenesis of *K. oxytoca* [14]. Therefore, the infection process of *K. oxytoca* is influenced by multiple mechanisms, and the specific pathogenic mechanisms still require further study.

Genome research is one of the most important directions in the field of modern microbiology, significantly contributing to our understanding of bacterial characteristics and the management of related diseases. Whole genome sequencing (WGS) technology, which integrates third-generation ONT [15] and second-generation Illumina methods, allows for the concurrent detection of multiple gene mutations with a high throughput and reduced time consumption [16]. This approach leverages the advantages of third-generation sequencing, characterized by long read lengths that effectively address assembly challenges such as repetitive sequences and structural variations, alongside the high accuracy of second-generation sequencing, which produces high-quality genomes through assembly without relying on a reference genome. These WGS methods offer comprehensive biological insights across entire genomes and can concurrently identify numerous pathogens in clinical samples [17]. The data generated through WGS can be processed with various bioinformatics tools [18], allowing for the assessment of the genome quality and the identification of the species and genotypes of infecting strains, as well as predictions regarding drug sensitivity, resistance, and epidemiological studies [19]. This information is of great value for early diagnosis, effective treatment, and epidemiological investigation. At present, there have been reports on the whole genome study of *K. oxytoca*, such as Campos [20], using Illumina and nanopore platforms for whole genome sequencing and species identification. Dickman [21] used Illumina Mi Seq for sequencing and compared this with the classification results obtained based on phenotypic techniques to determine the type of strain as *K. oxytoca*. Lo [22] used the Illumina Next Seq platform for sequencing and used the genomic results to perform an epidemiological analysis on the *K. oxytoca* collected in the experiment to evaluate its benefits for monitoring and understanding the outbreak of Enterobacteriaceae in the community. At present, the second-generation and third-generation mixed sequencing methods have not been reported in research. The related sequencing technology is mostly used for other common bacterial research areas such as research on *Mycoplasma* [23], *Salmonella* [24], and *Escherichia coli* [25] and tumor studies such as those on breast cancer [26], rectal cancer [27], etc.

The purpose of this study was to use ONT and Illumina sequencing techniques to assemble the genomic sequence of *K. oxytoca* and perform bioinformatics analyses to reveal its genomic structure and functional annotation. This study is of great significance for understanding the metabolic pathways of *K. oxytoca* and its mechanism of adapting to the environment and for analyzing its virulence genes and drug resistance genes, laying a foundation for future research. Furthermore, comparing the genome of KOHN1 with those of other distinct strains can aid in identifying significant drug targets and offer valuable insights for preventing disease outbreaks.

## 2. Materials and Methods

### 2.1. Strains

The KOHN1 strain of *K. oxytoca* was successfully isolated from the lung disease material of a Hainan black goat in this experiment. It was identified through colony-staining microscopy, a biochemical test, and 16S rRNA sequencing. A volume of 800 μL of goat-derived *K. oxytoca* solution was stored in 200 μL of 75% glycerol and stored in our laboratory’s ultra-low-temperature refrigerator.

### 2.2. Whole Genome Sequencing

Genomic DNA was extracted using the CTAB method [28]. After a quantitative quality inspection through NanoDrop microspectrophotometer detection (Nanodrop 2000, Thermo Fisher Scientific, Waltham, MA, USA), agarose gel electrophoresis (DYY-6C, Beijing Liuyi Instrument Factory, Beijing, China) and the use of a Qubit^®^ 2.0 Fluorometer (Life Technologies, Carlsbad, CA, USA), the ONT system and Illumina sequencing system were used for whole genome sequencing.

### 2.3. ONT and Illumina Sequencing

Following the standard protocol from ONT, the genomic DNA of the KOHN1 strain was fragmented using G-tubes (Covaris, Woburn, MA, USA) and subsequently end-repaired with the NEBNext FFPE DNA Repair kit (M6630, NEB, Ipswich, MA, USA) along with the NEBNext End Repair/dA-tailing Module reagents (E7546, NEB, Ipswich, MA, USA). Then, the fragments were ligated to adaptors to construct the sequencing library using NEBNext Quick Ligation Module reagents (E6056, NEB, Ipswich, MA, USA). After purification using the AMPure XP system (Beckman Coulter, Brea, CA, USA), library sequencing was performed on the long-read sequencing platform Oxford Nanopore PromethION (Oxford Nanopore Technologies, Oxford, UK) according to standard protocols. After that, the Illumina platform was used for calibration. Qualified genomic DNA was initially subjected to random sonication, followed by end repair, A-tailing, and adaptor ligation using the NEBNext^®^ ΜLtra™ DNA Library Prep Kit for Illumina (NEB, Ipswich, MA, USA) in accordance with the preparation protocol. DNA fragments ranging from 300 to 400 bp were then enriched through PCR. Finally, the PCR products were purified with the AMPure XP system (Beckman Coulter, Brea, CA, USA), and the libraries were assessed for size distribution using the 2100 Bioanalyzer (Agilent, Santa Clara, CA, USA) and quantified via real-time PCR. Genome sequencing was carried out on the Illumina NovaSeq 6000 sequencer employing paired-end technology (PE 150).

### 2.4. Genome Assembly

The sequence data obtained from ONT and Illumina sequencing were influenced by the characteristics of ONT sequencing, particularly its longer read lengths, which enhance the overall quality of genome assembly. The assembly process employs third-generation sequencing data, followed by the application of second-generation data through Fastp (version 0.20.0, parameter: -q 20 -u 50 -n 15 -l 50) to refine the assembly outcomes. After filtering the initial ONT and Illumina sequencing data, we acquired clean data. We first used Flye (version 2.9, parameter: -g 6 m -min_overlap 2000 -iterations 3) to assemble the filtered readings from scratch to produce a gapless overlap group. Pilon software (version 1.23, parameter: -mindepth 2 -mingap 10) was employed to refine the data, enhancing the accuracy and reliability of the assembly results. The initial ONT and Illumina sequencing data underwent filtering to generate clean data.

### 2.5. Genome Annotation and Bioinformatics Analysis

The bioinformatics analysis of the whole genome sequence included the identification of clustered regularly interspaced short palindromic repeats (CRISPRs), transposons, non-coding RNAs, repetitive sequences, coding sequences, genomic islands (GIs), and precursors. The identification of CRISPRs and transposons was carried out using CRISPRfinder (version 4.2.17) and TransposonPSI (version 20100822). The tRNA gene and rRNA gene were predicted using tRNAscan (version 1.3.1, parameter: -B) and RNAmmer (version 1.2, parameter: -S bac -m tsu, lsu, ssu), respectively. Cmscan (version 1.1.2, with the parameters --cpu 4 -E 1 × 10^−5^) was employed to compare the predicted sSRNA against the Rfam database. The dispersed repeat sequences were analyzed using RepeatMasker (version 4.0.5, parameter: -nolow -no_is -norna), and the coding sequences were identified utilizing the NCBI Prokaryotic Genome Annotation pipeline. TRF (version 4.09, parameter: 2 7 7 80 10 50 500 -m -d) was used to analyze tandem repeats. Gene islands were identified using IslandPath-DIMOB (version 1.0), while Phage_finder (version 2.0) was utilized to locate prophages.

### 2.6. Gene Function Annotation

Gene function analysis was performed using the databases of the Cluster of Orthologous Groups of proteins (COG) [29], Gene Ontology (GO) [30], the Kyoto Encyclopedia of Genes and Genomes (KEGG) [31], the Non-Redundant Protein Database (NR) [32], the Carbohydrate-Active enZYmes Database (CAZy), and TNSSs (Type N secretion systems, Types I–VII) [33]. The CARD (Comprehensive Antibiotic Research Database) [34] and the VFDB (Virulence Factors of Pathogenic Bacteria) [35] databases were used to predict and analyze the drug resistance genes and virulence genes in the genome. Details are shown in Table S1 and Figure S1.

### 2.7. Genome Visualization Analysis

For the assembled genome sequence, we integrated the predicted coding gene results and utilized Circos software (version 0.69-9) to create a circular map of the entire genome for the KOHN1 strain, so as to fully display the characteristics of the genome, such as the COG, KEGG, and GO functional classifications of the gene, the ncRNA prediction results, the distribution of the gene on the sense and antisense chains, the GC content, and so on. Finally, through the comprehensive display of the circle diagram, we could easily understand some basic characteristics of the KOHN1 strain genome.

### 2.8. Colinearity Analysis and SNP, InDels, and SV Statistics

The genomes of four human-derived model strains of *K. oxytoca* were selected (ATCC 13182, ASM381292, ASM2573217, and ASM3386047; see Table S2) as the target genomes. The KOHN1 genome served as the reference for conducting a collinearity analysis with the target genome. Firstly, Mummer (version 3.1, parameter: nucmer --maxmatch -c 100 -b 200 -l 50, ‘delta-filter -m -i 80 -l 100) was used to compare ATTC 13182, ASM381292, ASM2573217, ASM3386047, and KOHN1 to determine the large-scale collinearity between the genomes and to detect and analyze single nucleotide polymorphisms (SNPs). Subsequently, Syri (version 1.4, parameters: delta-filter -m -i 80 -l 100) was employed to assess the local positional arrangement of the samples and to check and analyze structural variations (SVs).

### 2.9. ANI, Gene Family Analysis, and Phylogenomic Tree Construction

Following the collinearity analysis, we employed PYANI (version 0.6.7, parameter: -m ANIb -g --workers 4) to compute the Average Nucleotide Identity (ANI) between the KOHN1 genome and the target genome. Subsequently, gene family analysis was performed on the target and reference genomes using the bidirectional best hit (BBH) criterion. This criterion stipulates that 80% of the shortest protein sequences exhibit a minimum of 40% amino acid similarity. Diamond (version 2.0.7, parameter: blastp -k 0 -f 6 -p 8 -e 1 × 10^−7^ --salltitles --more-sensitive) and Ortho MCL (version 1.4, parameter: mcl -I 1.5) were employed to compare and cluster the amino acid sequences, thereby obtaining homologous gene clusters and species distribution. Finally, single-copy gene families from both the KOHN1 and reference genome were selected to construct a phylogenetic tree using IQ-TREE (version 1.6.3, parameter: -nt 8 -m GTR+I+G -bb 1000 -st DNA) to study the evolutionary relationships among species, employing the maximum likelihood method to explore the phylogenetic relationships among strains.

### 2.10. Virulence Gene Detection

According to the reference for identifying the virulence gene primers of *K. oxytoca* (Table 1 and Table S3), related primers were synthesized. Using KOHN1 genomic DNA as a template, 10 virulence genes were detected through polymerase chain reaction (PCR) amplification. After the PCR (ETC811, Dongsheng Xingye Scientific Instrument Co., Ltd., Suzhou, China) amplification reaction, 1% agarose gel electrophoresis (Biowest Agarose; supplier: Beijing Mengyimei Business Center; brand: Biowest, Nuaillé, France) was used to determine the results.

## 3. Results

### 3.1. General Characteristics of KOHN1 Genome

**Figure 1:**
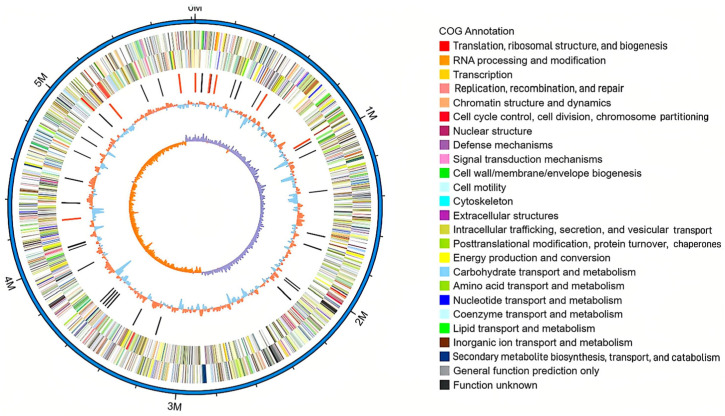
Whole genome map of KOHN1.The outermost circle illustrates the positional coordinates of the genome sequences, while the subsequent inner circles display various annotations and features, including the coding genes, COG database annotations, KEGG database annotations, GO database annotations, GC content, GC skew, and ncRNA. The GC skew was used to identify the starting and ending points on circular chromosomes, with both the window and step sizes configured to 1000. The outermost circle is the position coordinate of the genome sequence. From the outer circle to the inner circle, the order is the following: the positive chain gene, negative chain gene, ncRNA (black represents tRNA; red represents rRNA), GC content (red represents greater than the mean; blue represents less than the mean), and the GC skew (GC offset, used to measure the relative content of G and C, used to mark the starting point and end point in the circular chromosome; gC skew = (G − C)/(G + C); purple means greater than 0; orange means less than 0).

As shown in Table 2, the whole genome sequence of KOHN1 was 5,817,806 bp and the GC content was 55.14%. A total of 5227 genes were encoded by all genes. The total length of all genes was 5,087,988 pb, with an average length of 32,936.12 bp, and the total length of the coding region accounted for 87.46% of the whole genome. The number of copies of tandem repeats was 106, and the total length was 26,896 bp, accounting for 0.46% of the total length of the genome. There were 85 tRNA genes, 25 rRNA genes (including 8 23S rRNA genes, 8 16S rRNA genes, 9 5S rRNA genes), and 53 sRNA genes. There were eight gene islands and one prophage with a total length of 59,576 bp. The circular map of the KOHN1 genome was generated using Circos software, as illustrated in Figure 1 below.

### 3.2. Gene Function Annotation Results

**Figure 2:**
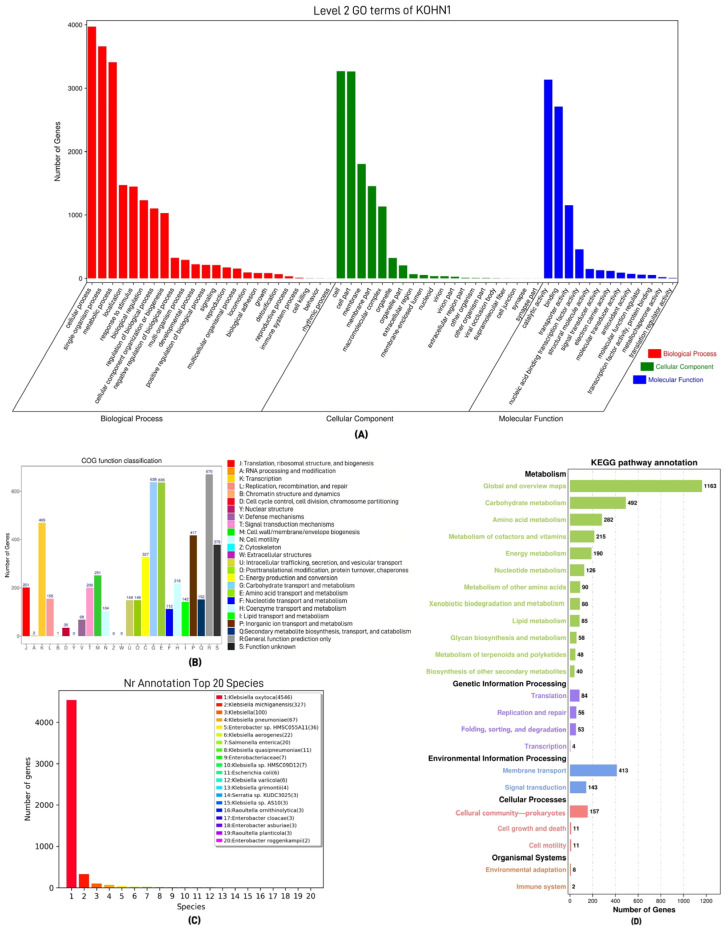
Analysis results of four different databases. (**A**) GO gene library annotation results. The *x*-axis illustrates the GO functional classification from the sample annotation, while the left *y*-axis shows the count of genes in the annotation. (**B**) NR gene library annotation results. The *x*-axis denotes the species ID, while the *y*-axis indicates the quantity of genes in the annotation. (**C**) COG gene library annotation results. The *x*-axis illustrates the functional classifications, shown in various colors, while the *y*-axis indicates the count of predicted genes associated with each classification. (**D**) KEGG gene library annotation results. The *x*-axis indicates the gene count, while the *y*-axis reflects the functional classifications from the KEGG database. The level 1 classifications are displayed in black font, and the level 2 classifications are shown in colored font. The figures on the bar graph represent the quantity of genes included in the annotation.

In the GO functional analysis (Figure 2A), the cellular processes and single-organism processes enriched 3969 and 3658 genes, respectively. In the COG analysis (Figure 2B), 4316 protein-coding genes were annotated into 25 categories from J to S, among which the pathway for general function prediction was the most enriched with 670 genes. Carbohydrate transport and metabolism enriched 638 genes, while amino acid transport and metabolism enriched 636 genes. In the NR analysis (Figure 2C), 5210 genes were annotated through a comparison with the NR database, with the highest number of genes annotated to *K. oxytoca*, totaling 4546. In the KEGG analysis (Figure 2D), the pathway with the highest enrichment was the Global and Overview maps at the metabolic level, which enriched 1163 genes. A total of 4102 genes were annotated in the four gene libraries (Figure S1).

The analysis indicated that the pathways with the highest gene enrichment in the COG, GO, and KEGG databases were predominantly metabolic processes. This underscores the significance of metabolism in the development of bacterial life, as it is essential for meeting the nutritional requirements necessary for growth and replication.

### 3.3. Results of CAZy Database Annotation

**Figure 3:**
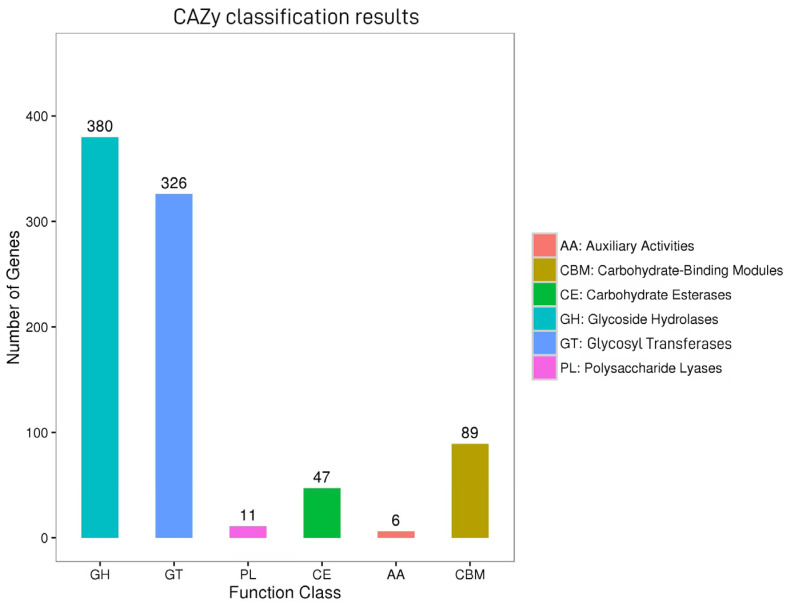
CAZy annotation results of KOHN1 gene. The *x*-axis denotes the classification of enzymes, while the *y*-axis indicates the count of genes that fall under each classification.

A total of 859 genes in the KOHN1 strain were annotated to the CAZy database (Figure 3), including 89 genes related to Carbohydrate-Binding Modules (CBMs), 47 genes related to Carbohydrate Esterases (CEs), 380 genes related to Glycoside Hydrolases (GHs), 326 genes related to Glycosyl Transferases (GTs), 11 genes related to Polysaccharide Lyases (PLs), and 6 genes related to Auxiliary Activities (AAs). Among the carbohydrate-active enzymes, Glycoside Hydrolases (GHs) represented the largest proportion, followed by GTs.

### 3.4. Results of Drug Resistance Gene Annotation

The CARD database was used to annotate all the genes of the KOHN1 strain. It was found that 448 genes in the KOHN1 strain were annotated to the CARD database. Seven genes were deleted under the condition of an 80% matching degree (Table S4). These drug resistance genes were mainly produced by encoding various enzymes and enhancing efflux. The analysis found that the strain contained one or more drug resistance genes related to β-lactams, tetracyclines, fluoroquinolones, cephalosporins, glycylcyclines, and fosfomycins corresponding to the multi-drug resistance phenotype of the KOHN1 strain.

### 3.5. The Results of the TNSS and Virulence Gene Analysis

**Figure 4:**
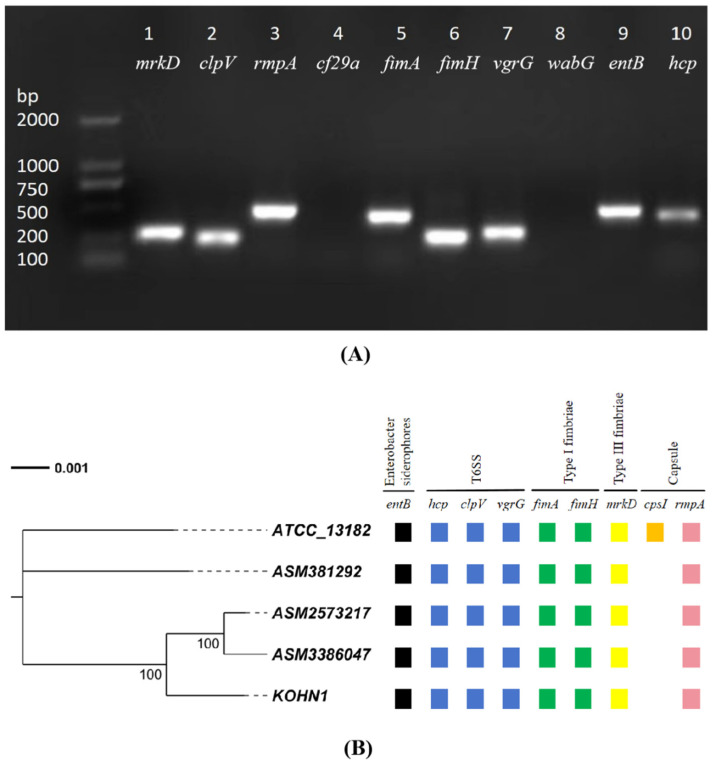
Virulence gene results. (**A**) PCR results of virulence genes of KOHN1 strain. M: DL2000 DNA Marker; 1: *mrkD*; 2: *clpV*; 3: *rmpA*; 4: *cf29a*; 5: *fimA*; 6: *fimH*; 7: *vgrG*; 8: *wabG*; 9: *entB*; 10: *hcp*. (**B**) Distribution of virulence factors of five strains.

In the TNSS analysis (Table S5), the KOHN1 strain harbored 14 T6SS effector proteins. Among these, *vgrG* and *hcp* serve as markers for T6SS activation, while *clpV* is a candidate key gene involved in the T6SS infection process. As a significant virulence gene in KOHN1, *clpV* also tested positive in subsequent PCR assays. In the comparative analysis with the VFDB database, a total of 146 genes were annotated, with the majority of annotations pertaining to virulence factor secretion systems and iron uptake (Table S6). Ten virulence genes were screened using VFDB analysis and detected through PCR. The results are shown below (Figure 4A). Eight virulence genes were detected, *fimA*, *fimH*, *entB*, *mrkD*, *rmpA*, *vgrG*, *clpV*, and *hcp*, while the PCR results for the *cf29a* and *wabG* genes were negative. Figure 4B shows the VFDB database annotation results of related virulence genes in five different *K. oxytoca* strains. All five strains carried the aforementioned eight genes; however, the annotation results for the *cps I* gene showed variations, with only the reference strain ATCC 13182 testing positive.

### 3.6. The Collinearity Analysis Results of the Five Strains

**Figure 5:**
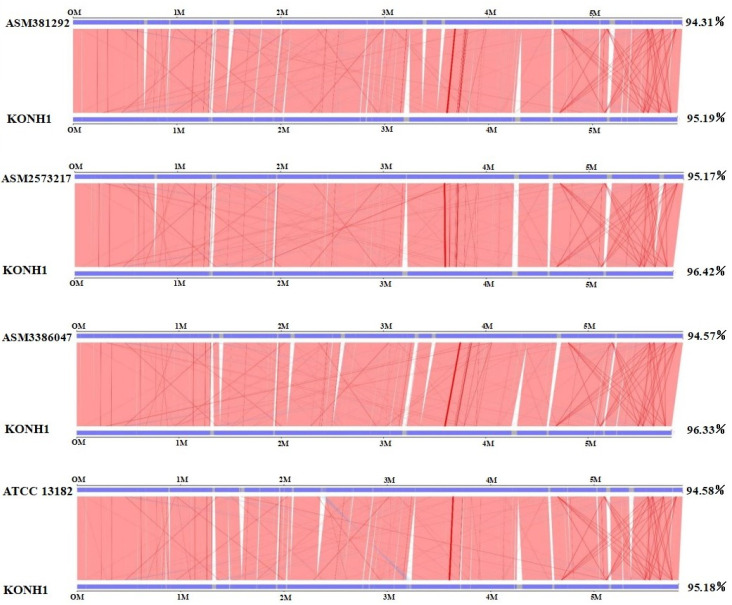
Parallel collinear result plot. The top axis illustrates the genome of the target species, while the bottom axis depicts the KOHN1 genome. The red line indicates the positive alignment of the corresponding regions, whereas the blue line represents the reverse alignment of those regions.

The collinearity results were statistically analyzed. On the basis of these results, a graph of parallel collinear results was drawn (Figure 5). According to the diagram, the alignment sequence region of the four target genes of KOHN1 accounts for more than 90% of the whole genome, indicating that the evolutionary distance between the five strains is relatively close. The strain with the closest evolutionary distance to KOHN1 is ASM3386047, and the strain with the farthest evolutionary distance from KOHN1 is ATCC 13182.

### 3.7. ANI Analysis and Phylogenetic Tree Analysis

**Figure 6:**
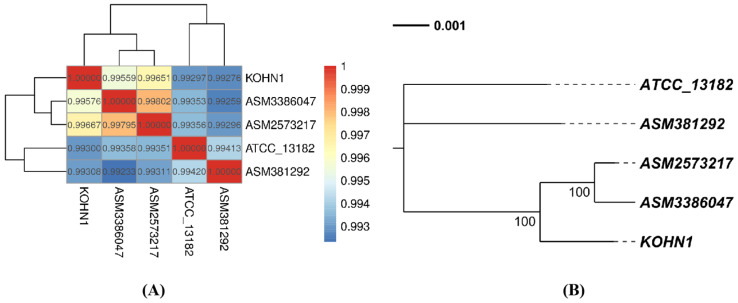
(**A**) ANI analysis heat map. (**B**) Phylogenetic tree based on characteristic genes. The phylogenetic tree depicts the evolutionary links and structural affiliations between different species. Each species is located at the tree’s tips, while the intersection points signify common ancestors. The length of the branches reflects the relative genetic distance among the species, and the numbers along the branches indicate their reliability.

Based on the collinear alignment, the ANI value was determined to create a heat map (Figure 6A). The results showed that the ANI value of ASM2573217 was the largest compared with KOHN1. This indicates that KOHN1 is closely related to ASM2573217. At the same time, the ANI value between ASM3386047 and ASM2573217 was the highest, indicating that there is a close relationship between them. Through the above diagram, we know that ASM2573217 has the closest relationship with KOHN1. The genetic distance between ASM3386047 and ASM2573217 was the closest. Due to the high similarity among the genomes of these five *K. oxytoca* strains, the genetic relationships between them cannot be accurately assessed through the analysis mentioned above.

Therefore, we further evaluated the genetic evolutionary distances between the strains using a phylogenetic tree. According to the results from the phylogenetic tree (Figure 6B), the genetic distance between KOHN1 and ASM3386047 was the smallest, followed closely by ASM2573217.

### 3.8. Gene Family Analysis and Data Results of SNPs, InDels, and SV

**Figure 7:**
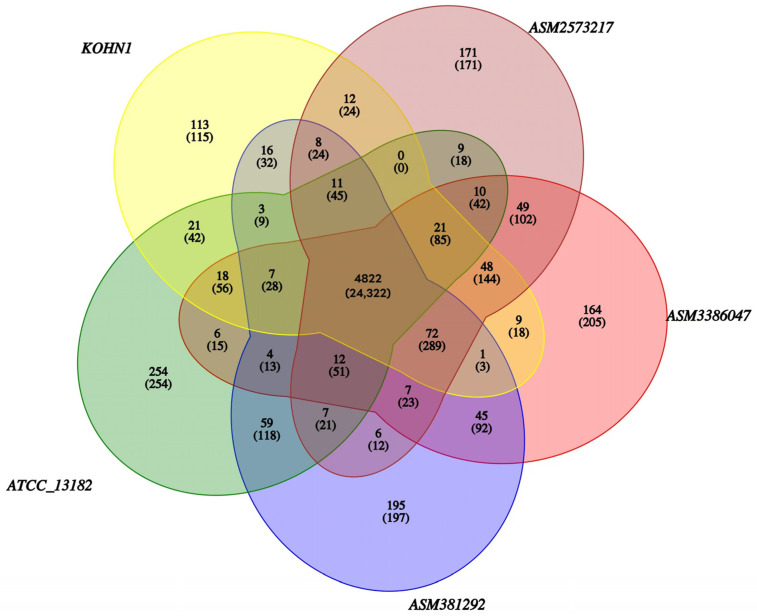
Homologous gene families of five strains of *K. oxytoca*. Each ellipse symbolizes a genome. The figure above each area indicates the count of gene families present in each species. The values in parentheses below denote the total number of genes within those gene families.

The whole genomes of five strains of *K. oxytoca* comprised a total of 26,570 genes, with the core genome consisting of 4822 genes. These 4822 genes are responsible for encoding the essential biological functions and key phenotypic traits of *K. oxytoca* (Table 3 and Figure 7). Among them, KOHN1 contains 113 specific genes, such as *PGB18_19815*, *PGB18_19945*, *PGB18_02255*, and so on.

Finally, we analyzed the quantitative relationship between SNPs, Insertions, Deletions, and SVs between KOHN1 and the target strain.

As shown in Table S7 and Figure S2, the genomes of KOHN1 and ASM2573217 exhibit the fewest SNPs, Insertions, and Deletions. Additionally, the number of SVs between KOHN1 and ASM3386047 is greater than that between KOHN1 and ASM2573217. Therefore, compared to KOHN1, ASM2573217 displays the least genomic variation, followed by ASM3386047.

## 4. Discussion

As of September 2024, the NCBI database included 1072 whole genome sequences of *K. oxytoca*, most of which were of low quality. Most of the early sequences are of low quality, at the level of Contingent or Scaffold. There are many species sources of *K. oxytoca* that have been included, but there is no goat source. In this study, the complete genome information of a goat-derived *K. oxytoca* was obtained for the first time, and the genome database information of *K. oxytoca* was expanded.

The whole genome size of KOHN1 was 5,817,806 bp, and the GC content was 55.14%. There were 2389 protein-coding genes in the whole genome of KOHN1 strain, and the total coding length of all genes was 5,087,988 bp. Simultaneously, seven potential CRISPRs were identified in *K. oxytoca* on the initial occasion. CRISPRs serve as a natural immune system in bacteria, allowing them to protect against phage sequences and foreign plasmids by effectively silencing these invading genetic elements [43]. In addition, compared with the average readings of the strains measured in Lo’s study [22], the genome size of this strain is higher than the average, which means that the strain may have a more complex structure and function of the organism, and has great potential in subsequent research.

CARD is a database based on antibiotic resistance ontology (ARO), which correlates information such as antibiotic modules, drug resistance mechanisms, and gene mutations. It provides data on drug resistance-related gene names and types of resistant antibiotics. In a previously reported antimicrobial susceptibility monitoring project for Europe [44], it was observed that the proportion of AMR in *Klebsiella* isolated from acute mastitis was low, with the highest resistance observed against tetracycline antibiotics. In this experiment, through the analysis of the CARD database combined with the previous experimental results in the laboratory, it was found that the strains of *K. oxytoca* isolated from goats were resistant to β-lactams, tetracyclines, fluoroquinolones, cephalosporins, aminoglycosides, fosfomycins, and other related drugs. The most common was the resistance to β-lactams. Differently from the report, the resistance of the goat-derived strain to cephalosporins and fluoroquinolones was observed in this study. It is speculated that it may be a multi-drug-resistant bacterium with high drug resistance.

The results of CAZy database analysis showed that the main enzymes of KOHN1 in the metabolic process were GH and GT. GH can decompose the glycosidic bonds between carbohydrate molecules and plays an important role in the hydrolysis and synthesis of polysaccharides in organisms. GT enzymes facilitate the attachment of activated sugars to various receptor molecules, including proteins, nucleic acids, and oligosaccharides, resulting in glycosylation products that serve numerous biological functions. In the study conducted by Vuillemin [45], it was noted that the GH family has emerged as a significant area of research in recent years, demonstrating a widespread presence in symbiotic intestinal bacteria and potentially participating in the degradation of animal glycans. In humans, it catalyzes the degradation of lactic acid-n-tetrasaccharide (LNT), an important component of human milk oligosaccharides that supports the development of healthy infant intestinal microbiota. We reasonably infer that this may be related to the pathogenesis of intestinal diseases caused by *K. oxytoca*.

In most studies on the number of *Klebsiella*, the T6SS is usually annotated [46]. In this study, the annotation results of the TNSS showed that the most important secretion system in this strain was also the T6SS. Research has demonstrated that the T3SS, T4SS, and T6SS are crucial for the pathogenicity of Gram-negative bacteria [47]. For *K. oxytoca*, some studies have mentioned that the bacteria will be killed in the host intestinal tract in a T6SS-dependent manner [48]. Therefore, this study concentrated on the T6SS. Research has shown that the T6SS, a transmembrane structure primarily found in Gram-negative bacteria, facilitates the direct transport of effectors from the cytoplasm to target cells or the extracellular environment through transmembrane channels. Pathogens exploit the T6SS to secrete effectors into extracellular spaces or host cells, thereby influencing critical processes such as immune responses and cell death, which can ultimately result in pathological reactions. In addition to the pathogenicity of bacteria, the T6SS also enhances their ability to adapt to various environments and engage in both intraspecific and interspecific competition. This is because the T6SS secretes some toxic proteins that can inhibit the growth of competitive bacteria and even kill them [49]. In this study, the main secreted protein of the T6SS was also an important virulence gene of KOHN1.

The VFDB is a comprehensive resource for studying the pathogenic factors of bacteria, including the pathogenic strains of Chlamydia and Mycoplasma. It provides essential evidence for investigating the mechanisms by which these pathogens invade host organisms. Combined with comparative genomics and other methods, the composition and genomic distribution of the virulence factors of different pathogenic bacteria were analyzed. The virulence factors used in the experiment have an important influence on *K. oxytoca*, in which *fimA* and *fimH* mainly encode type 1 fimbriae, which primarily function to enhance adhesion to host cells and tissues. These fimbriae have the ability to mediate adhesion, which is inhibited by soluble mannose-containing compounds *in vitro*, so they are often referred to as mannose-sensitive fimbriae [50]. Like *fim*, *mrkD* mediates adhesion by encoding type 3 biofilms [51]. Studies have shown that *fimA* and *mrkD* may regulate the formation of bacterial biofilms, which may be one of the mechanisms affecting the pathogenicity of *K. oxytoca*. As mentioned in Yang’s study [2], only a limited number of these genes have been experimentally investigated to assess their relationship with the virulence of *K. oxytoca*. In this study, PCR detection was used to determine the virulence relationship between these genes and *K. oxytoca*, which is an important virulence gene of the bacteria. *VgrG*, *hcp*, and *clpV*, as markers and key virulence genes of T6SS [52], also play an important role in the infection of *K. oxytoca*. According to Chen, these virulence genes, which are part of the T6SS, contribute to the development of bacterial biofilms, enhancing cell adhesion, promoting the survival and toxicity of bacteria, and enhancing the pathogenicity of *K. oxytoca*, which may be one of the pathogenic mechanisms of the bacteria. In addition, *hcp* is also regulated by iron ions, and *entB* acts as a siderophore gene that mediates iron uptake [53]. Since iron is involved in the biochemical metabolic reactions, electron transport, and other life activities of bacteria, it promotes the growth and reproduction of bacteria. Therefore, genes involved in iron uptake are also crucial to the pathogenicity of bacteria.

Through the comparative genomic analysis of KOHN1 and four other human-derived strains, we determined a high similarity between the goat-derived KOHN1 genome and the human-derived strains. The heat map and phylogenetic tree of the ANI analysis revealed the evolutionary and genetic relationship between the strains. Therefore, we infer that the infection route of the goat-derived isolate may be environmental infection. Based on the above results, we speculate that the pathogen may be transmitted between humans and animals. This provides a new perspective for the infection and spread of *K. oxytoca* and the consideration of biological health.

In this experiment, *K. oxytoca* was isolated from a Hainan black goat for the first time. Through genomic analysis, we could clarify its significant biological characteristics, including metabolic pathways, pathogenic genes, and drug resistance genes. This research is crucial for understanding the infection mechanisms of the bacteria, as well as for developing diagnostic methods and treatment strategies. Additionally, we obtained 113 unique genomes of *K. oxytoca* through comparative genomics, which included metabolism-related GT4, GT8, and GH3, potentially linked to its fermentation capabilities. Furthermore, studying its evolutionary relationship with human strains can enhance our understanding of the evolution and spread of *K. oxytoca*, as well as the differences among various strains. This knowledge is essential for monitoring and controlling the spread of pathogenic bacteria and guiding clinical treatment.

## 5. Conclusions

In this study, we obtained the whole genome sequence of a goat-derived *K. oxytoca* (KOHN1) and analyzed its fundamental biological functions. A total of 448 drug resistance genes were identified, and their multi-drug resistance to various antibiotics, including β-lactams and tetracyclines, was assessed in conjunction with previous experiments. A total of 146 virulence genes were obtained and virulence genes such as *VgrG*, *hcp*, *clpV*, and *fimA* were studied through PCR experiments. Through comparative genomics analysis, it was found that the strain contained 113 specific genes, including GH3, GT4, and GT8, related to carbohydrate metabolism. At the same time, this study also revealed the strain’s evolutionary relationship with human isolates. It provides a reference for future research on the drug resistance mechanism, transmission route, and pathogenic mechanism of *K. oxytoca*.

## Figures and Tables

**Table 1:** Virulence gene primer information.

Virulence Gene	Primer Sequences (5′-3′)	Annealing Temperature (°C)	Reference
*vgrG*	F: GCATCTTCCAACTCAACAC	55	Zhang A et al., 2021 [36]
	R: GTACACCAGCCCTTCTTC		
*fimA*	F: GCACCGCGATTGACAGC	61	Omer FH et al., 2022 [37]
	R: CGAAGGTTGCGCCATCCAG		
*fimH*	F: GCTCTGGCCGATACCACGACGG	55	Brisse S et al., 2009 [16]
	R: GCGAAGTAACGTGCCTGGAACGG		
*mrkD*	F: TAT TGGCTTAATGGCGCTGG	60	Brisse S et al., 2009 [16]
	R: TAATCGTACGTCAGGTTAAAGACC		
*hcp*	F: TGCTGAACGTGTTGAACATT	58	Hu YY et al., 2023 [38]
	R: TAACAGCACCTTTAGCAGTA		
*clpV*	F: CGAGGTCATTAAAGTTGCCCAATC	60	Luo G et al., 2019 [39]
	R: CGCCGAAACCGACATACCCT		
*cf29a*	F: GACTCTGATTGCACTGGCTGTG	51	Brisse S et al., 2009 [16]
	R: GTTATAAGTTACTGCCACGTTC		
*wabG*	F: CGGACTGGCAGATCCATATC	58	Lin WH et al., 2010 [40]
	R: ACCATCGGCCATTTGATAGA		
*entB*	F: ATTTCCTCAACTTCTGGGGC	56	Candan ED et al., 2015 [41]
	R: AGCATCGGTGGCGGTGGTCA		
*rmpA*	F: CGTATGAAGGCTCGATGGAT	60	Turton GF et al., 2010 [42]
	R: TGTGCACCATTTTTCATCAG		

**Table 2:** General characteristics of the KOHN1 genome.

Item	Number	Item	Number
Genome size (bp)	5,817,806	Number of rRNA genes	25
Total length of gene (pb)	5,087,988	Number of tRNA genes	85
Genome GC content (3%)	55.14	Number of sRNA genes	53
Average length of gene (bp)	32,936.12	Number of DNA elements	13
Number of genes	5227	Number of short interspersed elements	33
Number of gene islands	8	Number of long interspersed repeated sequences	27
Total length of coding region (%)	87.46	Total potential CRISPRs	7
Total length of gene island (bp)	263,489	Number of prophages	1
Number of tandem repeats	106	Total length of prophages (pb)	59,576

**Table 3:** Gene family analysis of five strains of *K. oxytoca*.

Strain Name	Total Genes Number	Number of Genes in the Family	Number of Specific Families	Unclustered Genes Number	Families
ATTC 13182	5311	5057	254	254	5010
ASM381292	5332	5161	171	171	5094
ASM2573217	5388	5255	164	133	5162
ASM3386047	5312	5118	195	194	5081
KOHN1	5227	5116	113	111	5071

## Data Availability

All data are presented in the article, and the raw sequence data reported in this paper have been deposited in the Genome Sequence Archive (Genomics, Proteomics & Bioinformatics 2021) in the National Genomics Data Center (Nucleic Acids Res 2022), China National Center for Bioinformation/Beijing Institute of Genomics, Chinese Academy of Sciences (GSA: CRA019326), which is publicly accessible at https://ngdc.cncb.ac.cn/gsa (accessed on 11 December 2024).

## Supplementary Materials

The following supporting information can be downloaded at https://www.mdpi.com/article/10.3390/genes16010013/s1: Table S1: Summary of genome function analysis of KOHN1 strain; Table S2: Reference genome information table; Table S3: Function of virulence gene; Table S4: CARD analysis results; Table S5: TNSS analysis results; Table S6: VFDB analysis results; Table S7: Statistical results of SNPs, InDels, and SVs between KONH1 and target strains; Figure S1: The Venn diagram of the annotation results of four databases; Figure S2: SNP matrix diagram.

## Abbreviations

The following abbreviations are used in this manuscript:

ONT	Oxford Nanopore Technologies
AMR	Antibiotic resistance
WGS	Whole genome sequencing
PCR	Polymerase chain reaction
COG	Cluster of Orthologous Groups of proteins
GO	Gene Ontology
KEGG	Kyoto Encyclopedia of Genes and Genomes
NR	Non-Redundant Protein Database
CAZy	Carbohydrate-Active enZYmes Database
TNSS	Type N secretion systems, Type I-VII
VFDB	Virulence Factors of Pathogenic Bacteria
CARD	Comprehensive Antibiotic Research Database
SNP	Single nucleotide polymorphisms
SV	Structural variation
ANI	Average Nucleotide Identity
GH	Glycoside Hydrolases
GT	Glycosyl Transferases
PL	Polysaccharide Lyases
AA	Auxiliary Activities
CE	Carbohydrate Esterases
CBM	Carbohydrate-Binding Modules
