# Supplementary figures and images for: Whole Genome Sequencing and Comparative Genomics Analysis of Goat-Derived Klebsiella oxytoca
Source: Genes (Basel). 2024 Dec 26;16(1):13. doi: 10.3390/genes16010013 (PMC11765384; doi:10.3390/genes16010013)

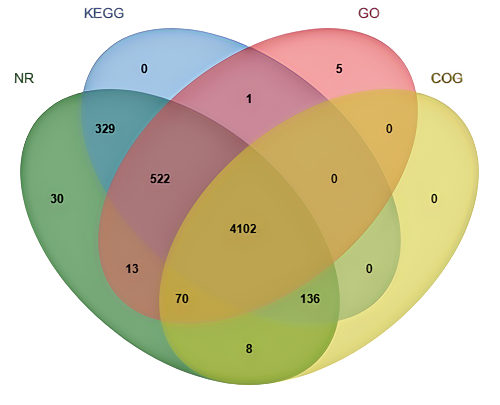

Supplement: Supplementary file 1 [file genes-16-00013-s001.zip › Figure S1.png]

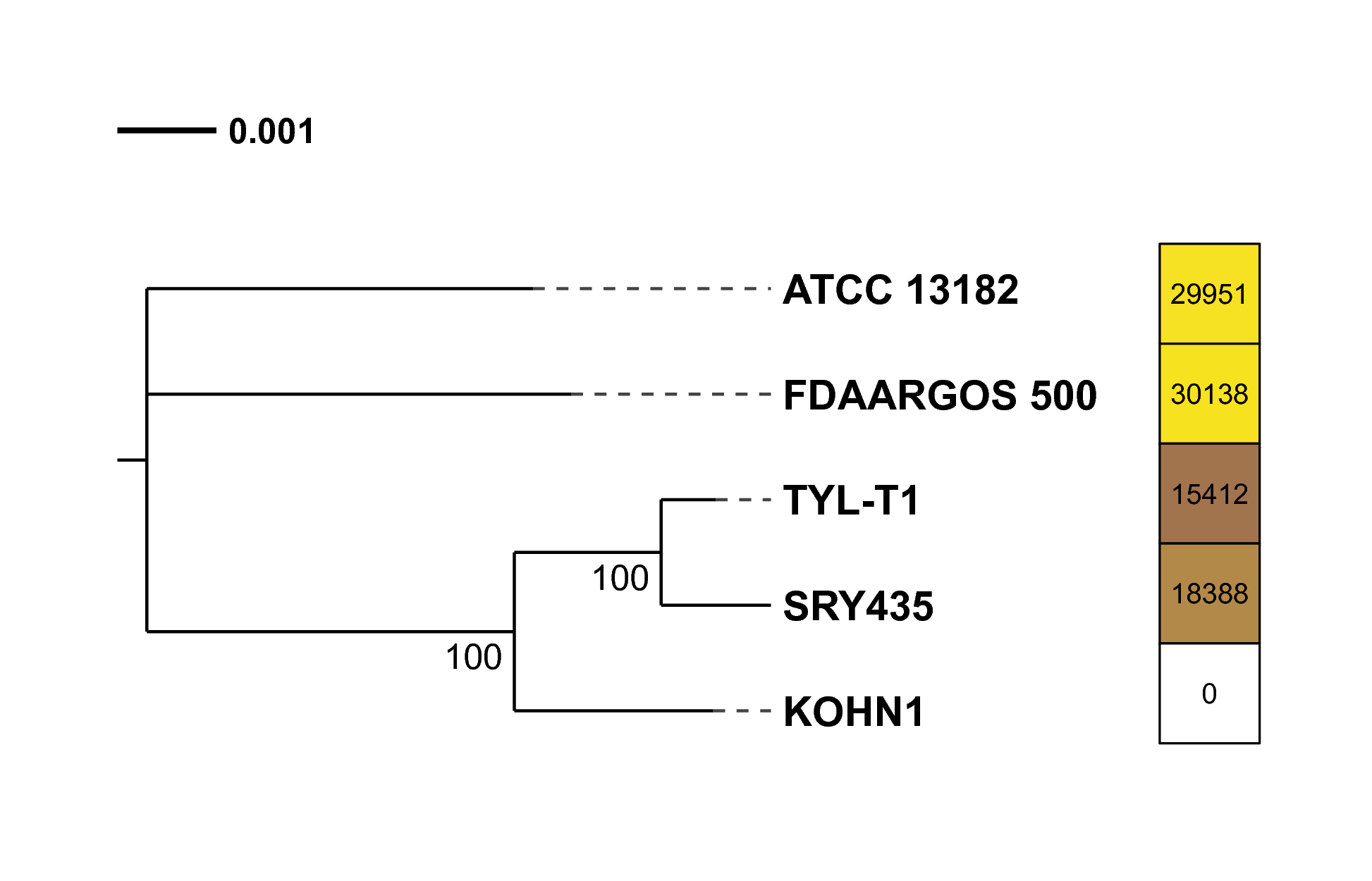

Supplement: Supplementary file 1 [file genes-16-00013-s001.zip › Figure S2.png]
